# Genetic counseling: development of requirements, contents, and quality management in Germany

**DOI:** 10.1515/medgen-2021-2056

**Published:** 2021-05-14

**Authors:** Friedmar R. Kreuz

**Affiliations:** Practice for Human Genetics Tuebingen, Paul-Ehrlich-Strasse 23, 72076Tuebingen, Germany

**Keywords:** genetic counseling, guidelines, psychosocial aspects, education process, quality management

## Abstract

To carry out quality management of genetic counseling, it is important to know what genetic counseling exactly means and who the players are. The term “genetic counseling” was first defined by Reed in 1947. It describes a communication process dealing with genetic facts and psychosocial aspects and is an education process, too. It has always been understood in the context of individual and family problems, and is unrelated to eugenics. In 1975 the Ad Hoc Committee of the American Society of Human Genetics published a more detailed description. With the development of new diagnostic techniques and methods in human genetics, the requirements of genetic counseling and its contents changed. Today a genetic counselor has to apply diagnostic, predictive, susceptibility, pharmacogenetic, carrier, prenatal, and preimplantation testing, as well as genetic screening. The German Human Genetic Examination Act (Genetic Diagnosis Act – GenDG) and national and international associations recommend to embed genetic testing into genetic counseling. Based on experiences of the author, some examples of pitfalls in genetic counseling are illustrated, as there are so many individual situations and requests that it seems impossible to carry out quality management. Nevertheless, the Commission for Quality in Genetic Counseling and Clinical Genetics of the Professional Association of German Human Geneticists started a pilot ring trial in 2018 with a given counseling situation. The task was to write the human genetics comment with the help of a checklist containing all issues necessary. The evaluation was conducted with the help of a catalogue of criteria which had been established beforehand and a score adjusted to the individual situation. The first genuine pilot trial was launched in 2020. It represents a possibility for quality management in genetic counseling.

## Introduction

Two years after the end of fascism and its inhumane eugenics theories and euthanasia methods, the American geneticist Sheldon C. Reed (1910–2003) coined the term “genetic counseling” to describe the process of helping families coping with medical and psychological effects of genetic diseases. Later he noted what this term means for him: “the term ‘genetic counseling’ occurred to me as an appropriate description of the process which I thought of as a kind of social work without eugenic connotations” [1]. He sharply distinguished the intent of eugenics (interests of the larger society) and counseling (interests of individual families).

In 1975 the Ad Hoc Committee of the American Society of Human Genetics (ASHG) defined the elements of an optimal genetic counseling process:

“Genetic Counseling is a communication process which deals with the human problems associated with the occurrence, or the risk of occurrence, of a genetic disorder in a family. This process involves an attempt by one or more appropriately trained persons to help the individual or family to

(1) comprehend the medical facts, including the diagnosis, probable course of the disorder, and the available management;
(2) appreciate the way heredity contributes to the disorder, and the risk of recurrence in specified relatives;
(3) understand the alternatives for dealing with the risk of recurrence;
(4) choose the course of action which seems to them appropriate in view of their risk, their family goals, and their ethical and religious standards, and to act in accordance with that decision; and
(5) to make the best possible adjustment to the disorder in an affected family member and/or to the risk of recurrence of that disorder” [2].

However, genetic counseling is more than a common dialog between the physician and the patient as seen in the paternalistic pattern of the patient–physician relationship. Moreover, genetic counseling conforms well with the deliberative pattern of this relationship and reflects the personal and familiar situation in its current and anticipated approach. The focus of genetic counseling is not only on the individual but also on the family. So, we have a special relationship between family and physician, which has not been described in previous patterns. This situation requires special training in communication and psychology and a feeling of intra-pair and intra-family relationships.

Ethical aspects imply that genetic counseling has to be voluntary, non-directive, non-active, focused on the individual or on the family, frank, and unbiased.

## Progress in genetic counseling

During the last 50 years changes not only in genetic diagnostic investigations but also in genetic counseling have occurred. There are a lot of new diagnostic methods in cytogenetics and molecular genetics which allow to diagnose more genetic diseases, not only in childhood but also in adulthood. In the early years the main question of the counselees, mostly a couple, was: “How high is the recurrence probability of the disease our child suffers from in the next pregnancy?” Certainly, this question will be asked today, too. However, today many genes are detectable and the question of individual persons is: “Where do I suffer from? Is it genetical? Is there a curative treatment?” Not always do the patients ask whether there is a recurrent probability for their children or other relatives.

Another challenge is the possibility to anticipate diseases which will develop later in life, like neurodegeneration diseases as Alzheimer’s, Parkinson’s, Huntington’s disease, or hereditary ataxias. Predictive genetic diagnosis requires a feeling for psychosocial aspects of getting a positive or negative result and for intra-pair and intra-familiar dynamics and dynamics between siblings. Predictive genetic diagnosis has ethical implications, too: There are reports about exploitations of the children by their parents and husbands by their wives in order to find out whether the other child would be ill. Siblings develop a so-called “survivor’s guilt” when they are not carriers of the pathogenic mutation [3]. And what about the situation, in the case of a dominant pattern of inheritance, where the healthy son wants to get diagnosed and the healthy father would not, because he is afraid of suffering from his father’s disease? Another problem is not only to cope with a bad result, but to cope with a good result of a predictive diagnosis as well. The author remembers a situation of a young woman who wanted to confirm her idea that she would become ill, because she had some characteristics of her mother, who had suffered from Huntington’s disease. The young woman adopted the role of her mother in housekeeping and protected her two brothers. She refrained herself from professional qualification and partnership. However, she received a good result and had a lot of problems to reorganize her life.

New fertilization techniques, prenatal methods, tumor genetics, and gene therapy require specific expert knowledge of all kinds of medicine and genetics. So genetic counseling is not only social work, as Reed described, but it becomes more and more an educational process. Time for communication of specific items is needed, and genetic counseling also deals with psychological, psychosocial, ethical, religious, legal, and ethnical aspects. Therefore, genetic counseling is more than talking about the recurrence probability and cannot take place in an ordinary setting, as a director of a university hospital explained to the author, pointing out that he is also able to perform genetic counseling – but has no time for all these additional aspects.

## Requirements to genetic counseling

The **German Human Genetic Examination Act (Genetic Diagnosis Act – GenDG)** determines patterns and contents of genetic counseling (Article 13, Section 3). Genetic counseling has to take place in an atmosphere comprehensible for everyone and possible medical, psychic, and social aspects connected with the question should be discussed to decide whether or not to undertake a genetic analysis and receive the result [4].

The German Gendiagnostik-Kommission (GEKO) ascertained that the contents of genetic counseling will be described through the guideline of the German Association of Human Genetics (S2k-Leitlinie der Deutschen Gesellschaft für Humangenetik [GfH]) [5]. The GEKO also agrees with the definition of the Ad Hoc Committee and describes common patterns for genetic counseling: information about objectives, volumes, motivations, and expectations. Genetic counseling has to be absolutely voluntary. Ethical aspects are to be considered, lay organizations have to be recommend, and relatives should be informed by the counselees about a genetic disease in the family and the possibility to take genetic counseling.

The **guideline of the GEKO** differentiates between the context of diagnostic, predictive, and prenatal genetic diagnosis. In case of a genetic diagnosis the counselor has to interpret the results of the investigation and their consequences for the counselee himself/herself and his/her relatives. In cases of multifactorial diseases, the counselor has to inform about manifestation, prevention, and therapy of the disease. Genetic counseling prior to predictive diagnosis has to deal with genetic and exogenic factors, specificity, sensitivity, predictive value, and false positive and false negative results. Prior to prenatal diagnosis the contents of genetic counseling are the basic risk, the hint of the claim of psychosocial counseling, the importance of possibilities the disease will occur, information on methods of investigations and their validities, possible consequences of prenatal genetic diagnosis, and risks of invasive methods to obtain test material. In case of a positive result of prenatal diagnosis the counselor has to inform about the phenotype of the child and the prognosis [5].

The abovementioned **guideline S2k “Humangenetics diagnosis and Genetic counseling”** describes values and content of genetic counseling more extensively. At the start of the genetic counseling process, the patient’s problems have to be defined and the counselor has to inform about purposes, value, and the following procedure. The counselor needs an input (actual anamnesis, autoanamnesis, pedigree information, earlier medical findings) to give an output about the disease with its whole clinical, genetical, and psychosocial aspects, especially indication, possibilities, validity, limits, and alternatives of genetic analyses. The counselor has to give support in the individual decision making (note: individual and religious values, psychosocial situation, ethical and technical limitations) and in coping with the impact of genetic information. The right not to know and to revoke has to be emphasized [6].

Genetic counseling is defined by the **World Health Organization (WHO)** as “[...] the process through which knowledge about the genetic aspects of illnesses is shared by trained professionals with those who are at an increased risk of either having a heritable disorder or of passing it on to their unborn offspring. A genetic counselor provides information on the inheritance of illnesses and their recurrence risks; addresses the concerns of patients, their families, and their health care providers; and supports patients and their families dealing with these illnesses” [7].

The WHO, the Organisation for Economic Co-operation and Development (OECD), the European Society of Human Genetics (ESHG), the ASHG, and other associations like the American Heart Association accentuate the necessity of genetic counseling related to genetic analysis and demand an improving access to genetic counseling services [8], [9].

Referring to the definition of the Ad Hoc Committee of the ASHG in 1975 the Task Force of the **American National Society of Genetic Counselors (NSCG**) developed a new definition of genetic counseling: “Genetic counseling is the process of helping people understand and adapt to the medical, psychological and familial implications of genetic contributions to disease. This process integrates the following:

– Interpretation of family and medical histories to assess the chance of disease occurrence or recurrence.
– Education about inheritance, testing, management, prevention, resources and research.
– Counseling to promote informed choices and adaptation to the risk or condition” [10].

For improving the quality of genetic analysis, EuroGentest, in co-operation with ESHG, recommends genetic counseling, too. In the paper of EuroGentest a definition of genetic counseling is given analogous to the definition of the Ad hoc Committee. Moreover, EuroGentest describes eight types of genetic analysis situations and the need for genetic counseling:

(1) *Diagnostic testing:* Because of the involvement of relatives this situation is different from other medical investigations. EuroGentest suggests that a pretest genetic counseling may not be necessary in every case but but genetic counseling is suggested in the case of a bad result (posttest counseling). However, the author advocates another opinion: A pretest counseling seems necessary for the decision making process in order to find out (i) the implications of a positive result on relatives, cure and prognosis of the disease, and the probability of recurrence.
(2) *Predictive testing:* Pre- and posttest genetic counseling has to be offered. However, there should be an interval of at least four weeks between the first contact and the sampling to reconsider the decision and to conclude on an insurance contract and other important issues. Counseling sessions and psychological support should be offered more often. This is the author’s opinion as well as that of the German lay organization of Huntington’s disease (Deutsche Huntington-Hilfe e. V.) [11].
(3) *Susceptibility testing:* This means genetic testing of a marker to detect a risk for a multifactorial condition. When the result of the test has implications of a risk assessment, treatment or prevention pre- and posttest counseling becomes essential.
(4) *Pharmacogenetic testing:* This testing of a genetic susceptibility for adverse drug reactions or for individualized drug treatment needs counseling by a specialist. Genetic counseling is necessary as soon as genetic implications for the test person or his or her relatives are found.
(5) *Carrier testing:* In the cases of carrying out genetic analysis such as segregation analysis or heterozygous tests in healthy persons, this is equal to predictive testing. Pre- and posttest genetic counseling has to be offered and is absolutely necessary, because there may be a high probability for the offspring to suffer from a disease. In Germany the GenDG subsumes carrier testing as predictive testing. According to the author’s experience genetic counseling in these cases should be given sensitively and with psychological understanding. Carrying a pathogenic mutation for a recessive disorder does not make a person ill, as some counselees believe.
(6) *Prenatal testing:* This genetic investigation will be carried out during pregnancy. Pre- and posttest counseling and psychological support should be offered.
(7) *Preimplantation genetic diagnosis* (*PGD*): In the author’s opinion genetic counseling should not only be offered as pre- and posttest counseling. Genetic counseling and psychological support are necessary during the whole process before *in vitro* fertilization, while genetic analysis can be performed after the result is communicated.
(8) *Genetic screening:* Genetic screening is offered to (a part of) the general population. The probability of a selected group, for instance newborns, to suffer from a genetic disease is not so high. However, pre- and posttest information is necessary like in other medical examinations. But if, as a result of screening, the person might belong to a high-risk group, genetic counseling should be offered [12].

**Figure 1:**
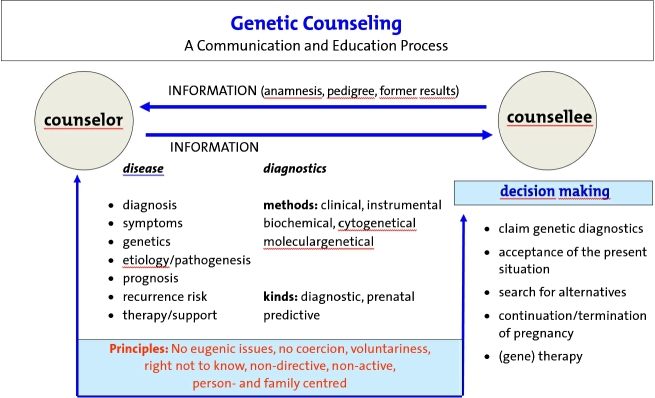
Genetic counseling as a communication and education process and its principles.

EuroGentest established recommendations for genetic counseling (see Appendix A) [12]. Figure 1 summarizes the essential aspects and principles of genetic counseling as a communication and education process.

## Quality management and special counseling situations

The former paragraphs dealt with the modes and contents of genetic counseling. The problem is how to guarantee high quality, which is necessary more than ever.

Genetic counseling is an individual person- and family-centered communication process. Every counseling situation is unique. Although every counselor has its own pattern and is trained to react on specific situations, there must be a consistent approach. The reflection of the counseling process is the written summary, the counselor’s so-called human genetics comment (“Humangenetische Stellungnahme,” “Humangenetisches Gutachten”) or, briefly, the counseling comment (“Beratungsbrief”).

Certainly, the counseling comment contains, in case it is written for more counselees (for instance a couple), genetic information of persons. In the case of a couple the wife or husband is not allowed to know some genetic facts of his or her partner. Maybe, both appear for counseling, but in the case of marital separation and a new partner, genetic data are not safe. This is an ethical and legal problem and can just be solved by writing two comments; each contains only the facts and data for one individual. Another problem, the author remembers, was the case of a supposed autosomal recessive disorder a child suffered from; the parents asked for the recurrence probability. They were told 25 %. Surprisingly, the father was asked to go outside, and the mother told that he is not the father of the child and that he never should know this. She asked, how high is the recurrence probability now? It was not easy to give a written answer comprehensible for both. Another example was a family with cancer with five children. After genetic diagnosis the lab phoned, that there must be an error because two of them disagree with the result. Asking the mother, she told that there was a marital crisis and the neighbor had been very friendly. How to describe this in the counseling comment?

**Figure 2:**
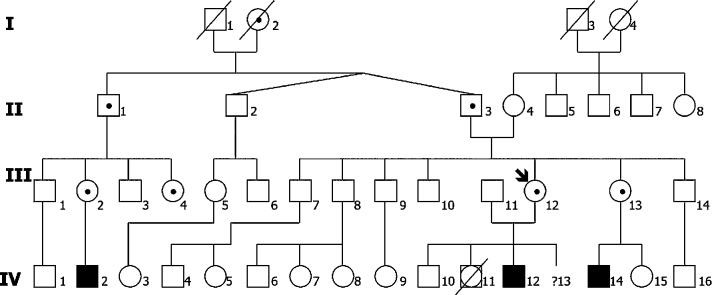
Pedigree of a family with fragile X syndrome. The pregnant counselee (III-12 and arrow) only asked for the teratogenic effects of hormonal contraception during pregnancy. Her mentally retarded son (IV-12) might suffer from congenital toxoplasmosis, not from a genetic disease. She reported on mental retardation in her nephew (IV-14) and in her cousin’s son (IV-2). The assumed diagnosis of fragile X syndrome was confirmed with the help of a molecular diagnosis. She herself, her sister (III-13), the mutual father (II-3), her uncle (II-1), cousins (III-2 and III-4), and her grandmother (I-2) are obligatory carriers of an *FMR1* premutation. Square = male; circle = female; filled square = affected male; point in symbols = carrier; crossed out symbol = deceased person; crossed out square and circle = stillbirth with unknown gender; Roman numeral = generation; Arabic numeral = order in the generation.

The importance of a pedigree analysis, the consequences for the counselees and relatives, and the documentation in the counseling comment illustrates another example (Figure 2). A pregnant woman (III-12) was offered genetic counseling by her gynecologist. She became pregnant although she had taken contraceptive pills regularly. The question was, do the hormones injure the development of the embryo? During the counseling she told about her mentally retarded child because of congenital toxoplasmosis (IV-12). Plotting the extensive pedigree she reported about mental retardation of the son of her sister (IV-14) and her cousin (IV-2). The reason of the mental retardation was unknown. Not all things the counselees tell us are reliable and the analysis of the pedigree suggested an X-linked mental retardation, for instance fragile X syndrome, with her father (II-3) and uncle (II-1) as carriers. Genetic analysis confirmed the assumption and prenatal diagnosis was offered and done. So, the initial question of possible teratology effects changed to a true genetic problem.

But, how we can guarantee and verify a high quality of genetic counseling? Indeed, there are some recommendations for improving genetic counseling and educational programs in genetic counseling and its accreditation but there is no quality management (EuroGentes [12], ACGC – Accreditation Council for Genetic Counseling [13], Lea DH [14]).

The commission supervising for quality in genetic counseling was established by the Professional Association of German Human Geneticists (Qualitätskommission Genetische Beratung und Klinische Genetik des Berufsverbandes Deutscher Humangenetiker – BVDH) in order to develop methods for quality management of genetic counseling. In 2005, a first questionnaire-based survey was carried out to get basic information about the quality of structures of counseling units in Germany [15]. In a second step (2005–2009) the commission launched two further surveys and asked to drop counseling comments to six problems in genetic counseling: (i) prenatally diagnosed trisomy 21 (Down syndrome), (ii) recurrent miscarriages, (iii) hereditary cancer disease in a family, (iv) fragile X syndrome in a boy (presumed and verified), (v) counseling and diagnosis of a couple with an unfulfilled desire to have a child before carrying out IVF/ICSI, and (vi) presentation and diagnosis of a child with indeterminate mental retardation and dysmorphic signs without a detected cause. This allowed to get an overview about the range of comments. As a result of this investigation a catalogue of criteria was established and a checklist with issues, necessary for both standardization and quality management, was created. The catalogue (see Appendix B) aimed at the definitions of genetic counseling and guidelines mentioned above [16].

In 2018 the commission started a pilot survey. As a result, the catalogue of criteria was adjusted. In this survey two problems for genetic counseling were described: (i) hereditary breast and ovarian cancer in a family and (ii) counseling of parents of a child with cytogenetically verified Down syndrome. The 19 participants of the pilot survey had to write genetic comments. The valuation was carried out with the help of the catalogue of criteria. On the basis of these criteria, credits were assigned. This way all comments were scored and when the score was above 60 %, the examination was passed. All participants passed the pilot survey. However, some noticeable problems occurred:

– missing reason for counseling and problem;
– missing information about accompanying persons;
– missing detail about generations in the pedigree;
– missing information about other ill relatives and the implication for the counselee;
– missing implications for relatives;
– describing of diseases and facts which are irrelevant for the actual situation;
– missing information about support and self-help organizations.

In 2020, the first true ring trial “Genetic Counseling” was launched. In this ring trial the participants had to comment on two problems: (i) fertility disorder in a man with deletion of the AZFc region and heterozygous mutation in the gene *CFTR* and (ii) histologically verified colon cancer in the counselee and a positive family history of colon cancer. The results are expected in 2021.

The commission holds the view that evaluation and quality management of genetic counseling is only possible by scoring human genetics comments. Such ring trials should be carried out every year. To take part in ring trials like laboratories allows to preserve a good standard and to improve the quality of communication in genetic counseling.

## Appendix A

Recommendations for genetic counseling established by EuroGentest [12].

1) General recommendations: Genetic counseling
  i) has to be an integral part of the genetic testing process;
  ii) cannot be compulsory;
  iii) should be offered and strongly recommended;
  iv) has to be provided or supervised;
  v) should be given in a well-understood language; and
  vi) should involve persons who are not able to give informed consent.
2) Pretest genetic counseling
  a) has to inform about
    i) the purpose of the test;
    ii) up-to-date, reliable descriptions of symptoms and the natural history of the disease;
    iii) prospects of prevention or early diagnosis and treatment;
    iv) the inheritance pattern;
    v) the probability of the disease in the counselee’s situation;
    vi) available reproductive choices;
    vii) reliability and limitations of the test concerned;
    viii) the possible psychological impact and other consequences of the test result;
    ix) privacy and confidentiality of the results;
    x) possible consequences related to disclosure to third parties (insurance companies, employers);
    xi) the absence of coercion; and
    xii) the right to know and the right not to know;
  b) has to discuss about
    i) possible uncertainties due to a present lack of knowledge; and
    ii) the need to inform relatives; and
  c) has to offer
    i) further genetic counseling sessions;
    ii) consultations with a psychologist;
    iii) contact with a social worker;
    iv) contact to lay support organizations;
    v) written materials and reliable internet addresses;
    vi) assistance in decision making;
    vii) ample time for decision making; and
    viii) a written summery.
3) Posttest genetic counseling
  a) has to offer
    i) follow-up contacts;
    ii) consultation with a psychologist;
    iii) contact with a social worker;
    iv) contact to lay support organizations; and
    v) a written summery of the test result;
  b) has to repeat point (1);
  c) has to discuss
    i) implications to the individual and their relatives; and
    ii) a strategy to inform relatives; and
  d) has to hand out written material.

## Appendix B

Catalogue of criteria to score human genetics comments.

General criteria
  volume
  segmentation (with headlines)
  understandability for all recipients
  terminological and objective failures
Formal criteria
  recipient(s)
  name, date of birth, address of the counselee
  dates of counseling
  reason of counseling
  specific problem
  summary
  offer of queries
Substantial criteria
  anamnesis of the counselee
  pertinent former medical reports
  family history
  declaration of the number of generations in the pedigree
  declaration whether there is consanguinity
  pertinent medical reports of relatives
  documentation of the pedigree in the human genetics comment
  results of investigations carried out by the counselor him or herself or induced
  declaration of further approach
  declaration of support (lay organizations, psychological support)
Subject-specific criteria
  declaration of clinical aspects of the disease
  declaration of genetic or non-genetic etiology (diagnosis or differential diagnosis)
  interpretation and assessment of anamnesis and family history
  considering of all important facts or explanation why issues are not important
  interpretation and assessment of medical reports
  declaration of the specific genetic probability
  declaration of general probabilities
  declaration and explanation of decision options for counselees
  implications for relatives
Specific criteria in the mentioned constellation
  history of pregnancy and former medical reports
  clinical investigation when occurred
  schedule of clinical findings, done by the counselor
  schedule of non-genetic findings
  schedule of genetic findings
